# Diagnostic accuracy of transthoracic echocardiography for the identification of proximal aortic dissection: a systematic review and meta-analysis

**DOI:** 10.1038/s41598-023-32800-4

**Published:** 2023-04-11

**Authors:** Bayu Sutarjono, Abrar Justin Ahmed, Anna Ivanova, Brandon Buchel, Joseph Rauscher, Alanna O’Connell, Jeremy Riekena, Aluko Gift, Matthew Kessel, Ekjot Grewal

**Affiliations:** Department of Emergency Medicine, Brookdale University Hospital and Medical Center, 1 Brookdale Plaza, Brooklyn, NY 11212 USA

**Keywords:** Cardiovascular diseases, Echocardiography, Ultrasonography

## Abstract

This systematic review and meta-analysis evaluated the performance of transthoracic echocardiography (TTE) for diagnosis of proximal aortic dissections based on the identification of specific sonographic features. A systematic literature search of major databases was conducted on human studies investigating the diagnostic accuracy of TTE for proximal aortic dissection. The study followed the Preferred Reporting Items for Systematic Reviews and Meta-Analyses statement. The quality of studies was evaluated using Quality Assessment of Diagnostic Accuracy Studies 2 tool. Data were gathered for the following sonographic findings: intimal flap, tear, or intramural hematoma; enlargement of aortic root or widening of aortic walls; aortic valve regurgitation; or pericardial effusion. Sensitivity, specificity, diagnostic odds ratio, number needed to diagnose values, and likelihood ratios were determined. Fourteen studies were included in our final analysis. More than half of the included studies demonstrated low risk of bias. The identification of intimal flap, tear, or intramural hematoma was shown to have an exceptional ability as a diagnostic tool to rule in proximal aortic dissections. TTE should be considered during the initial evaluation of patients presenting to the emergency department with suspected proximal aortic dissection. Positive sonographic findings on TTE may aid in rapid assessment, coordination of care, and treatment of individuals awaiting advanced imaging.

## Introduction

Acute proximal aortic dissection is an uncommon but life-threatening condition with a unique set of diagnostic challenges. It follows two classification systems: Stanford^1^ and DeBakey^2^. Stanford type A lesions involve the ascending aorta, whereas the DeBakey system accounts for pathology affecting ascending and descending aorta (type I) or only the ascending segment (type II).

Computed tomography angiography (CTA) is considered the reference standard for noninvasive diagnosis of proximal aortic dissection. It enables the visualization of the entire aorta and distinguishes among the different types of acute aortic syndromes^3^. However, this technique is not always viable at all institutions, carries radiation burden, and can result in substantial delays in treatment. Acute proximal aortic dissection is a time-sensitive emergent condition in which mortality increases by 1% each subsequent hour without intervention, with a mortality rate of 36–72% by 48 h^4^. For those who undergo surgical intervention, the 48 h mortality rate is 4.4%^5^.

TTE is a readily accessible bedside tool in the emergency and critical care settings. Given its rapidity, availability, portability, and safety, it is the ideal imaging technique for the initial evaluation of patients with suspected proximal aortic dissection. Many clinical studies have been conducted to investigate the diagnostic performance of TTE for the detection of proximal aortic dissection. However, there are conflicting results in these studies driven by the variability of scanning strategies. Therefore, a systematic review of literature was performed in the present study to evaluate the diagnostic accuracy using strategies that scan for individual or combination of sonographic features in patients presenting with acute proximal aortic dissection. Specifically, the search strategies include the identification of an intimal flap, tear, or intramural hematoma; the enlargement of the aortic root or widening of the aortic walls; the existence of aortic valve regurgitation; the presence of pericardial effusion; or the systematic scan for any of the aforementioned categories.

## Methods

We performed a systematic review and meta-analysis of studies that compared the performance of echocardiography when used to assess type A aortic dissection. This study followed the guidelines in the “Cochrane Handbook for Systematic Reviews of Diagnostic Test Accuracy.”^6^ This study was registered with PROSPERO International Prospective Register of Systematic Reviews (registration number CRD42022356272).

### Eligibility criteria

Studies were considered eligible for this systematic review and meta-analysis if they fulfilled the following criteria: (1) any randomized or non-randomized human studies investigating type A or ascending dissection using TTE, with the reference standard clearly defined; and (2) both prospective and retrospective studies were eligible. We excluded studies when they met one of the following criteria: (1) experimentation with animals; or (2) reviews, commentary, and case reports.

### Search strategy

A standardized search was done in PubMed, OVID MEDLINE, Embase, and Web of Science, using the following search terms found in titles and abstracts: (ultrasound OR echocardiography OR sonography) AND ("type A" OR ascending OR proximal) AND (aortic OR aorta) AND dissection. The search was done on September 5, 2022 with no language restrictions.

### Study selection

Two authors screened and selected studies independently based on the criteria described above, with disagreements resolved by consensus together with a third author. Studies identified from different databases were de-duplicated after screening. Articles that passed the initial screening were reviewed for the full text. Studies with data available on true negative, true positive, false negative, and false positive results were included for the meta-analysis. This study followed the Preferred Reporting Items for a Systematic Review and Meta-analysis of Diagnostic Test Accuracy (PRISMA)^7^. The checklist can be found in the Supplemental Material.

### Data collection process and data items

Two authors gathered pertinent data from the included studies, with disagreements resolved by a third author. Individual data of sample size, number of true positive, true negative, false positive, and false negative results per imaging modality from each included study were extracted. If only partial information was available, outcomes were calculated using results from the reference standard. Based upon how findings were presented in the included studies, datasets were nominally categorized according to sonographic findings. These findings included the (1) intimal flap, tear, or intramural hematoma, (2) enlargement of aortic root or widening of aortic walls recorded at least 4 cm in size or greater, (3) aortic valve regurgitation, (4) pericardial effusion, or (5) the systematic search of any of the four aforementioned sonographic features. Dichotomous findings of positive or negative for each were compared against the standard reference. Information on the reference standard, population, location, study design, and technical aspects of the ultrasound machine, and characteristics of the sonographer and retrospective reviewer of ultrasound clips were also retrieved.

### Risk of bias

The quality of each study was appraised with the Quality Assessment of Diagnostic Accuracy Studies 2 (QUADAS-2) tool, structured into patient selection, index test, reference standard, and flow and timing, structured as a list of 13 items and and qualified as “yes,” “no,” or “unclear” for an individual study. Each domain was evaluated for the risk of bias and the first three in terms of applicability. The answers were used to judge whether the risk of bias and concern for the applicability of the research is low, high, or unclear. Two reviewers independently judged the quality of each study, with disagreements resolved by consensus with additional input from a third.

### Synthesis of results

Subgroup analyses for all categories of sonographic features were conducted. Sensitivity, specificity, diagnostic odds ratios, number needed to diagnose, and likelihood ratios with the associated 95% confidence intervals were calculated from true negative, true positive, false negative, and false positive cases with a 0.5 continuity correction for zero events. All *P* values were two-sided, and any *P* value < 0.05 was considered statistically significant. Forest plots were generated for individual studies according to sonographic features with summary estimates for each category and overall estimates. Heterogeneity was assessed, whereby *P* < 0.05 for Cochran's Q and Higgin's I^2^ > 0.500 indicate significant heterogeneity. Since variability among studies was not only due to sampling error, but also to variability in the population of effects, the random effects model using the inverse variance method was used if heterogeneity is high, which was determined by comparing the Cochrane Q to the critical value for its respective degree of freedom as found in a chi-square distribution, and subsequently I^2^ > 0.500 using the fixed effect model. Summary receiver operating characteristics (SROC) curve plotting sensitivity (true positive rate) against 1-specificity (false positive rate) was generated. Area under the SROC curve (AUC) served as proxy for diagnostic accuracy, whereby AUC > 0.900 indicate excellent diagnostic accuracy. All statistical analyses were performed using Microsoft Excel (version 2021).

### Role of funding source

There was no funding source for this study.

## Results

### Description of studies

**Figure 1:**
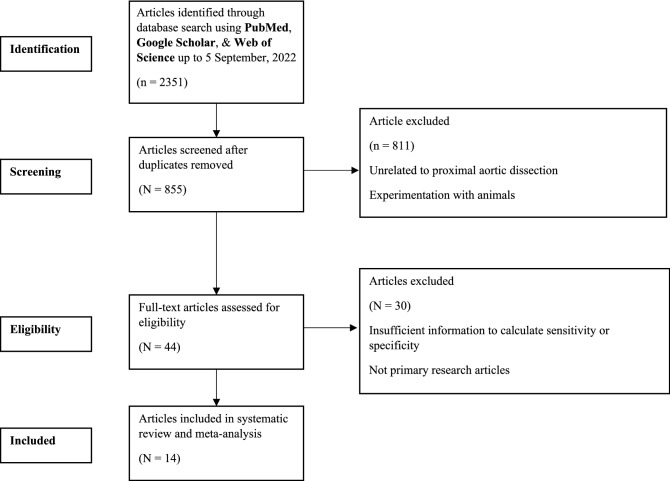
Study profile.

2351 studies were identified in our research. After assessing the titles and abstracts, 44 full texts were screened, as shown in Fig. 1. On the basis of our selection criteria, 30 of those studies were excluded. Therefore, 14 studies^8–21^ met our inclusion criteria. Characteristics of the included studies are found in Table 1.

**Table 1:** Characteristics of included studies.

Author	Study design, setting	N	Country	Mean age	Female	Reference standard	Sonographic features
Asouhidou^8^	Sing. Pro.	23	Greece	55	18.0%	CT/CTA	N/A
Carrel^9^	Sing. Pro.	61	Switzerland	57	23.0%	Surgery/autopsy	Flap/tear/hematoma Aortic root/wall dilatation Pericardial effusion Aortic regurgitation
Enia^10^	Sing. Pro.	46	Italy	62	4.3%	CT/CTA	Flap/tear/hematoma Aortic root/wall dilatation
Khandheria^11^	Sing. Retro.	67	USA	N/A	N/A	CT/CTA, surgery/autopsy	Flap/tear/hematoma Aortic root/wall dilatation
Nazerian^12^	Sing. Pro.	281	Italy	70	10.3%	CT/CTA	Flap/tear/hematoma Aortic root/wall dilatation Pericardial effusion Aortic regurgitation
Nazerian^13^	Mult. Pro.	839	Brazil, Germany, Italy, Switzerland	62	35.6%	CT/CTA, transesophageal echocardography, MRI, surgery/autopsy	Flap/tear/hematoma Aortic root/wall dilatation Pericardial effusion Aortic regurgitation Penetrating aortic ulcer
Panchavinnin^14^	Sing. Pro.	16	Thailand	N/A	N/A	N/A	Flap/tear/hematoma
Pare^15^	Mult. Retro.	16	USA	61	31.3%	CT/CTA	Flap/tear/hematoma Aortic root/wall dilatation Pericardial effusion
Sobczyk^16^	Sing. Retro.	172	Poland	59	26.4%	CT/CTA, surgery/autopsy	Flap/tear/hematoma Aortic root/wall dilatation Pericardial effusion Aortic regurgitation
Thurau^17^	Sing. Retro.	512	Germany	61	33.0%	Surgery/autopsy	Pericardial effusion Aortic regurgitation
Tokuda^18^	Sing. Retro.	24	Japan	75	62.5%	CT/CTA	Pericardial effusion
Wang^19^	Sing. Pro.	72	China	58	25.0%	CT/CTA	Flap/tear/hematoma
Wu^20^	Mult. Retro.	265	China	48	23.4%	CT/CTA, surgery/autopsy	Pericardial effusion Aortic regurgitation
Zhan^21^	Sing. Retro.	361	China	50	24.4%	CT/CTA, MRI	N/A

*Sing*. single-center, *Mult*. multi-center, *Pro*. prospective, *Retro*. retrospective, *CT* computed tomography; *CTA* computed tomography angiography; *MRI* magnetic resonance imaging.

The studies were published from 1989 to 2021. The majority of the studies took place in Europe^8–10,12,13,16,18^, followed by Asia^14,19–21^. Six studies were conducted prospectively, for a total of 1315 patients^2,3,5,6,14,19^. Participation of all studies were from the adult population.

### Sonographic views investigated

The most common sonographic feature investigated was the presence of intimal flap, tear, or intramural hematoma^9–16,19^, followed by aortic root or wall dilatation^10,12,13,15,16^. Pericardial effusion^12,13,15,17,18,20^ and aortic regurgitation^12,13,16,18,20^ were the least studied features. The majority of studies^8–13,15,21^ investigated multiple sonographic features indicative of aortic dissection.

### Risk of bias assessment

**Figure 2:**
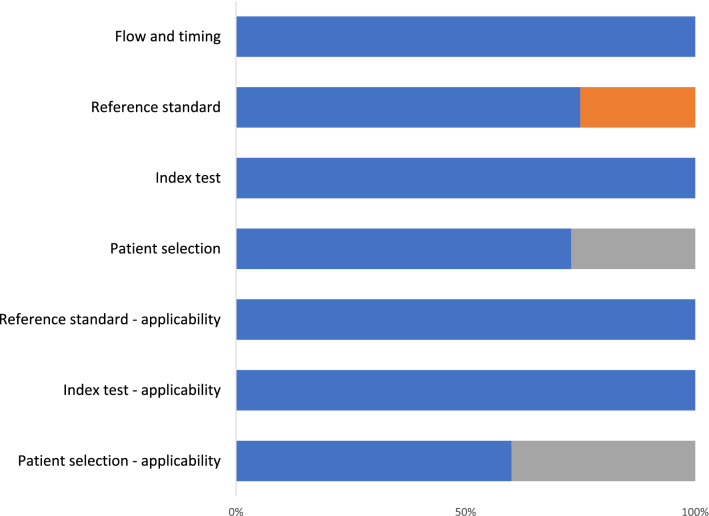
Quality assessment of diagnostic accuracy studies 2 (QUADAS-2) finding per domain for included studies in the systematic review. Blue represents low level of bias, grey represents high level of bias, while orange represents unclear level of bias.

Table 2 depicts the risk of bias assessment using QUADAS-2 tool, with visual representation in Fig. 2. Only one study^12^ had high risk of patient selection bias due to non-consecutive or non-random selection of patients. This study used convenience sampling. Six studies^8,9,11–13,15^ had high risk of flow and timing bias, as these studies did not receive the same reference standard. These studies varied confirmation of the proximal aortic dissection by using CT with contrast or CTA^8,9,11–13,15,21^, transesophageal echocardiogram^12,13^, MRI or magnetic resonance angioraphy^13,15,21^ chest xray^21^, or surgery or autopsy^11,12,15^.

**Table 2:** Risk of bias assessment performed with the Quality of Assessment of Diagnostic Accuracy Studies-2 (QUADAS-2) tool.

	Risk of bias										Applicability concerns		
	Domain 1: Patient selection			Domain 2: Index text		Domain 3: Reference standard		Domain 4: Flow and timing			Patient selection	Index test	Reference standard
	1. Was a consecutive or random sample of patients enrolled?	2. Was a a case–control design avoided?	3. Did the study avoid inappropriate exclusions?	1. Were the index test results interpreted without knowledge of the reference standard?	2. If a threshold was used, was it pre-specified?	1. Is the reference standard likely to correctly classify the target condition?	2. Were the reference standard results interpreted without knowledge of the results of the index?	1. Was there an appropriate interval between index test and reference standard?	2. Did all patients receive the same reference standard?	3. Were all patients included in the analysis?			
Asouhidou^8^	Yes	Yes	Yes	Yes	Yes	Yes	Yes	Yes	No	Yes	Low	Low	Yes
Carrel^9^	Yes	Yes	Yes	Yes	Yes	Yes	Yes	Yes	No	Yes	Low	Low	Yes
Enia^10^	Yes	Yes	Yes	Yes	Yes	Yes	Yes	Yes	Yes	Yes	Low	Low	Yes
Khandheria^11^	Yes	Yes	Yes	Yes	Yes	Yes	Yes	Yes	No	Yes	Low	Low	Yes
Nazerian^12^	No	Yes	Yes	Yes	Yes	Yes	Yes	Yes	No	Yes	Low	Low	Yes
Nazerian^13^	Yes	Yes	Yes	Yes	Yes	Yes	Yes	Yes	No	Yes	Low	Low	Yes
Panchavinnin^14^	Yes	Yes	Yes	Yes	Yes	Yes	Yes	Yes	Yes	Yes	Low	Low	Yes
Pare^15^	Yes	Yes	No	Yes	Yes	Yes	Yes	Yes	No	Yes	Low	Low	Yes
Sobczyk^16^	Yes	Yes	Yes	Yes	Yes	Yes	Yes	Yes	Yes	Yes	Low	Low	Yes
Thurau^17^	Yes	Yes	Yes	Yes	Yes	Yes	Yes	Yes	Yes	Yes	Low	Low	Yes
Tokuda^18^	Yes	Yes	Yes	Yes	Yes	Yes	Yes	Yes	Yes	Yes	Low	Low	Yes
Wang^19^	Yes	Yes	Yes	Yes	Yes	Yes	Yes	Yes	Yes	Yes	Low	Low	Yes
Wu^20^	Yes	Yes	Yes	Yes	Yes	Yes	Yes	Yes	Yes	Yes	Low	Low	Yes
Zhan^21^	Yes	Yes	Yes	Yes	Yes	Yes	Yes	Yes	No	Yes	Low	Low	Yes

### Sensitivity/specificity

**Figure 3:**
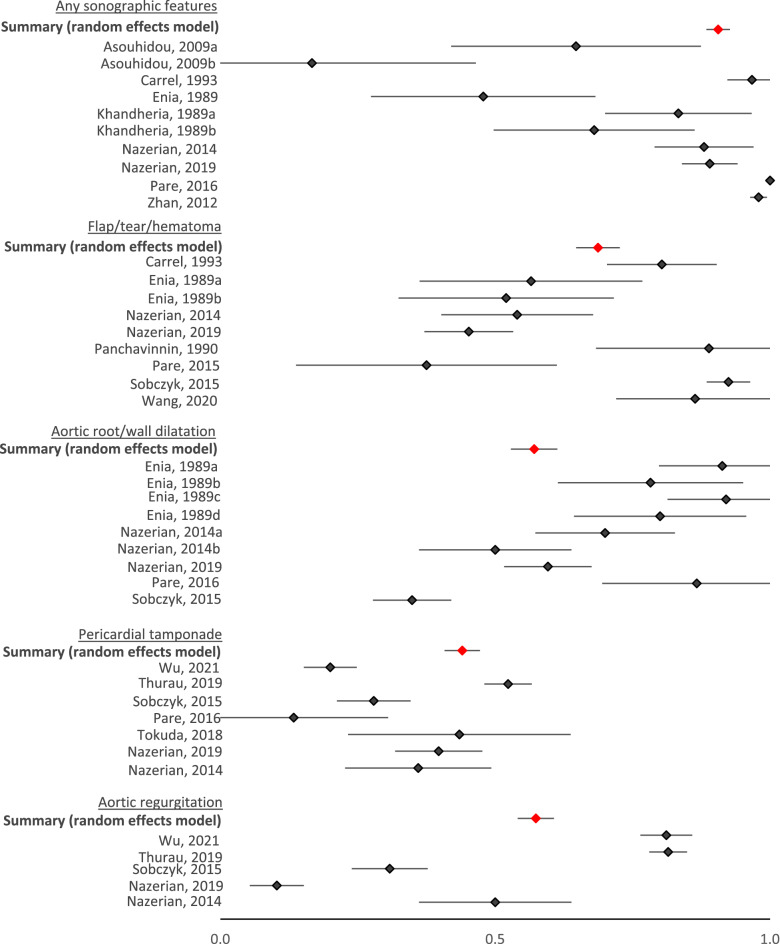
Forest plots of sensitivities of all studies. Lowercase letters denote subcategories of studies taking place within the article.

**Figure 4:**
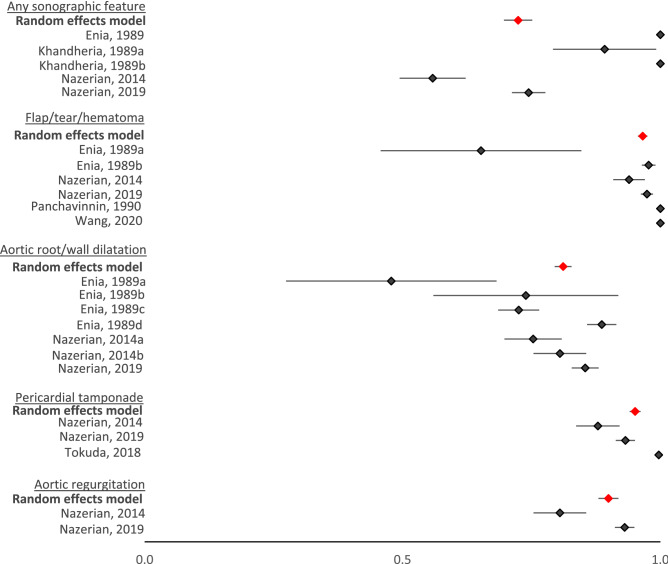
Forest plots of specificities of all studies. Lowercase letters denote subcategories of studies taking place within the article.

A total of 9602 sonographic examinations were conducted using TTE with positive results confirmed predominantly by CT, surgical operations, or autopsies as reference standard. Forest plots of all studies are presented in Figs. 3 and 4. Sensitivity ranged from 10.3 to 100.0% across all studies. The highest sensitivity parameter was the identification of any sonographic feature of aortic dissection on TTE (i.e. intimal flap, tear, or intramural hematoma; aortic root or wall dilatation; pericardial effusion; and/or aortic regurgitation) at 90.6% [95% CI 88.4–92.7%], followed by the identification of intimal flap, tear, or intramural hematoma at 68.7% [95% CI 64.7–72.7%]. The overall sensitivity for all studies was 62.4% [95% CI 60.8–64.0%]. Specificity ranged from 47.8 to 100.0% for all studies. The feature with the highest specificity was the identification of intimal flap, tear, or intramural hematoma at 96.6% [95% CI 95.6–97.5%], followed by the identification of pericardial effusion at 95.1% [95% CI 94.0–96.2%]. The overall specificity for all diagnostic tests was 87.5% [95% CI 86.7–88.3%].

### Diagnostic odds ratio

The diagnostic odds ratio from individual studies ranged from 1.5 to 249.2 for the different sonographic features. Pooled diagnostic odds ratios are presented in Table 3. The feature with the highest diagnostic odds ratio was the identification of intimal flap, tear, or intramural hematoma at 61.9 [95% CI 61.5–62.2], followed by the identification of any sonographic feature at 25.2 [95% CI 25.0–25.5]. Aggregate diagnostic odds ratio for all noted sonographic investigations of all studies carried was 11.6 [95% CI 11.5–11.7].

**Table 3:** Pooled diagnostic odds ratio, number needed to diagnose, and likelihood ratios.

	Pooled diagnostic odds ratio (95% CI)		Pooled Number needed to diagnose (95% CI)		Positive likelihood ratio (95% CI)		Negative likelihood ratio (95% CI)	
Any sonographic feature	25.2	(25.0–25.5)	1.59	(1.30–1.87)	3.28	(3.18–3.39)	0.30	(0.19–0.42)
Intimal flap, tear, or intramural hematoma	61.9	(61.5–62.2)	1.53	(1.20–1.87)	20.04	(19.53–20.56)	0.05	([− 0.22]–0.32)
Aortic root or wall dilatation	5.7	(5.5–5.9)	2.62	(2.41–2.82)	3.03	(2.87–3.18)	0.33	(0.22–0.44)
Pericardial effusion	15.3	(15.0–15.5)	2.56	(2.29–2.82)	9.00	(8.60–9.39)	0.11	([− 0.11]–0.33)
Aortic regurgitation	12.0	(11.8–12.3)	2.11	(1.86–2.37)	5.70	(5.46–5.95)	0.18	([− 0.01]–0.36)

### Number needed to diagnose

Pooled values for the number needed to diagnose for TTE ranged from 1.53 to 2.62, with all subgroups showing similar impact. All results are shown in Table 3.

### Likelihood ratios

Pooled values for positive likelihood ratio ranged from 3.03 to 20.04, while pooled values for negative likelihood ratio ranged from 0.05 to 0.33. All categories were statistically significant (two-tailed *P* > 0.05). Results are found in Table 3.

### Cochran's Q and Higgin's I^2^

Using the random effects model, the Cochran's Q for TTE was 0.000 with 35 degrees of freedom (two tailed, *P* > 0.05). Subsequently, the Higgin's I^2^ for overall results was 0.000, indicating low heterogeneity. Similarly, subgroup analysis revealed low heterogeneity for each sonographic feature.

### AUC

**Figure 5:**
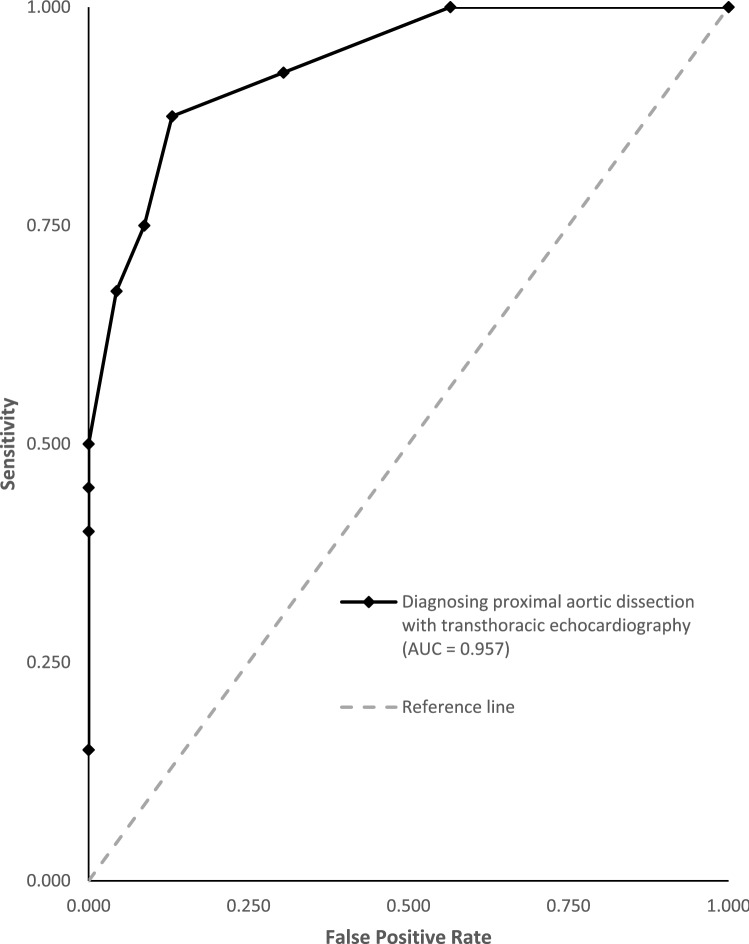
Overall SROC curve plotting sensitivity (true positive rate) against 1-specificity (false positive rate).

Overall evaluation for TTE produced outstanding discriminating ability, as shown in Fig. 5. Each sonographic feature had an AUC of 1.000, with the overall datasets scoring 0.957.

## Discussion

To our knowledge, this is the first systematic and meta-analysis examining the diagnostic performance of TTE for the identification of proximal aortic dissection. In summary, the overall sensitivity and specificity when all five search strategies are considered are 62.4% and 87.5% respectively. However, our analysis suggests that the identification of an intimal flap, tear, or intramural hematoma has exceptional diagnostic utility to rule in proximal aortic dissections, given its high specificity (96.6%), high odds ratio (62), high positive likelihood ratio (20.04), low negative likelihood ratio (0.05), and low number needed to treat (1.53).

In contrast, we discovered that the systematic assessment of each sonographic feature has the potential to lower suspicion for proximal aortic dissections considering its reasonably high sensitivity (90.6%), high diagnostic odds ratio (25), low likelihood ratio (0.30), and low number needed to treat (1.58). Future studies should therefore investigate the development of pretest probability in the manner similar to the PERC and Wells score^22^, where the identification of a particular group in combination with negative ultrasound findings following the systematic assessment of any sonographic feature can efficiently rule out proximal aortic dissections with a high degree of confidence.

Based on these results, it is reasonable to recommend TTE for the initial evaluation of patients presenting to the emergency department with suspected proximal aortic dissection. There are also clear advantages of ultrasound in comparison to the gold standard of CTA: the imaging is more accessible as it can be used be used by a single physician^23^, and assessments are performed bedside, thereby eliminating the risk of delay transporting patients to the radiology suite^23^. Within minutes, TTE can identify sonographic findings consistent with aortic dissection, warranting expedited advanced aortic imaging or transfer to specialized centers^13^. Furthermore, exposure to radiation and contrast load are eliminated^24^, and is therefore suitable for a broader subset of patients in which CTA may present complications. Its serial use is also advantageous for identification of delayed complications secondary to aortic dissection such as the development of pericardial tamponade requiring emergent drainage. Finally, although hospitals in resource-rich populations have access to advanced imaging, this is not the case for health centers in resource-limited regions, such as rural towns or countries with many isolated or austere villages. In these locations, it is therefore imperative that patients with proximal aortic dissections are rapidly transported to centers with imaging and treatment capabilities^25^. Therefore, a means of accurately identifying patients with proximal aortic dissections is important for rural USA, Tanzania, or Australian Outback.

This systematic review showed many strengths with regards to the selection of the 14 studies. Nearly half of the sample size of all studies (47.7%) were from prospective studies, while 40.6% were from multi-center studies. The representation of the centers were distributed around the world between Europe (7), Asia (5) and the Americas (3), with results gathered over more than three decades of research. Furthermore, the QUADAS-2 tool showed relatively low risk of bias from all studies, while there was no evidence of significant heterogeneity among included studies and among different types of sonographic features (Cochrane Q = 0.000, I^2^ = 0%).

Our study had few limitations of note. In particular, 8 of the 14 studies had a sample size below 100, which were traditionally regarded as small studies. These studies could have led to an overestimated effect size as a result^26^. Furthermore, there were variances in half of the studies with regards to the reference standard, as these studies used a combination of modalities, such as CT, MRI, TEE, surgery, and autopsy, to identify proximal aortic dissection. However, these diagnostic variations are all likely accurate and reliable and may be explained by the condition of the patient and course of presentation. Finally, only one study noted the experience required to perform TTE^12^, and only two studies provided information of the ultrasound system^10,12^. It is therefore reasonable to assume that TTE is highly dependent on the examiners’ techniques and requires caution in interpretation.

In conclusion, we show that there is diagnostic value in the utilization of TTE for the diagnosis of proximal aortic dissection. In summary, the identification of an intimal flap, tear, or intramural hematoma effectively rules in the disease with a high level of confidence, while the absence of any sonographic feature following a systematic TTE scan greatly lowers suspicion. We suggest that employing this TTE search strategy can provide pivotal information early on during the patient's presentation and should be considered as a routine tool for triage and assessment for proximal aortic dissection.

## Supplementary Information

Supplementary Information.

## Data Availability

The original data generated in the current study are available from the corresponding author.
